# Disseminated Coccidioidomycosis in an Immunocompetent Male Who Lived in an Endemic Region in the Remote Past: A Case Report

**DOI:** 10.7759/cureus.25249

**Published:** 2022-05-23

**Authors:** Priyal Agarwal, Rakesh Gami, Abdul F Osman, Si Yuan Khor, Issa Haddad

**Affiliations:** 1 Internal Medicine, Michigan State University, East Lansing, USA

**Keywords:** diagnostic microbiology, endemic mycoses, disseminated coccidioidomycosis, pulmonary coccidioidomycosis, medical mycology, challenging diagnosis, endemic fungi

## Abstract

Coccidioidomycosis is an endemic illness suspected in patients who live in or have recently traveled to an endemic area. Disseminated disease is less frequent and is almost always seen in the presence of risk factors such as immunosuppression. We present a case of disseminated coccidioidomycosis with a delayed presentation in a young immunocompetent male. The patient developed symptoms two years after migrating from the endemic region of Mexico. He presented with fever, cough, and shortness of breath for two weeks. Chest imaging revealed left-sided consolidation and pleural effusion. Empyema was ruled out by thoracentesis. The patient did not improve with antibiotics for community-acquired pneumonia. A comprehensive microbiological workup for bacterial, viral, mycobacterial, and fungal etiologies, including cultures of several specimens of sputum, pleural fluid, blood, bronchoalveolar lavage, serological tests (initial), and transbronchial lung biopsy, was nondiagnostic. The patient continued to have fever and shortness of breath despite the escalation of antibiotic coverage to broad-spectrum. The patient underwent an open surgical lung biopsy, and the diagnosis of coccidioidomycosis was ultimately established by histopathological examination of lung and pleural specimen which showed spherules of *Coccidioides *sp. The patient developed worsening headaches, a lumbar puncture was done and cerebrospinal fluid revealed coccidioidal antibody which confirmed meningeal dissemination. Human immunodeficiency virus/acquired immunodeficiency syndrome or other immunosuppressed state was not identified in the patient. Notably, the second set of antibody titers collected two weeks after the initial negative set of titers returned strongly positive. The patient was started on fluconazole but did not show clinical improvement and was switched to amphotericin B. Subsequently, the patient improved and was discharged on lifelong oral fluconazole with close outpatient clinical and serological monitoring. He has had no signs of relapse during the last 20 months.

## Introduction

Coccidioidomycosis is commonly known as valley fever, desert rheumatism, or San Joaquin valley fever. It is caused by *Coccidioides immitis* and *Coccidioides posadasii*, which are dimorphic fungi endemic to dry desert environments of the western hemisphere such as the southwestern United States (central valley of California, Arizona, parts of New Mexico, and Texas), northern Mexico, and certain parts of central and south America. It is acquired by inhaling the fungal spores. Spores are present in the soil of the endemic areas and can become airborne when soil is disrupted such as during construction, farming, windstorms, or earthquakes. Thus, people with certain occupations such as farming or construction work or those involved in outdoor recreational activities are at an increased risk. Cases are seen mostly in endemic areas but can be imported to non-endemic areas by travel. The spectrum of disease manifestation range from asymptomatic infection to severe disseminated disease. The incubation period is around 7-21 days. Most infections (approximately 60%) are asymptomatic. Symptomatic infections include primary pulmonary, progressive pulmonary, and disseminated disease [1]. Most pulmonary infections are self-limited. Patients usually develop a cough, fever, shortness of breath, myalgia, arthralgia, fatigue, and rash. These symptoms typically resolve within a few weeks to months. Patients who have underlying risk factors such as an immunocompromised state, age >65 years, diabetes mellitus, or smoking are at risk of developing severe pulmonary complications (5-10%) such as cavitary lesions, fibrosis, or disseminated disease (less than 1%) which most commonly involves the skin, bone, and meninges. Laboratory tests are non-specific, and elevated erythrocyte sedimentation rate (ESR) or eosinophilia may be seen. Chest imaging is also non-specific and can appear similar to community-acquired pneumonia. Reticulonodular pattern, hilar or mediastinal adenopathy, and pleural effusions can also be present on chest imaging. These findings are more commonly seen with progressive or chronic pulmonary illness [2]. The diagnosis of coccidioidomycosis is usually established by fungal culture or by visualization of *C. immitis* spherules in body fluids and biopsy specimens or by serological tests [3]. Polymerase chain reaction (PCR) can also be done but is not widely available. Here, we present a rare case of disseminated coccidioidomycosis in a young immunocompetent male who lived in the endemic region in the remote past. This case report also examines the complexity of the diagnostic approach and management of disseminated coccidioidomycosis.

## Case presentation

A 39-year-old previously healthy Hispanic male presented with shortness of breath, dry cough, and fever for two weeks. He reported associated intermittent headaches and unintentional weight loss of around 10 pounds in the previous two months. The patient had migrated from Mexico to the United States about two years prior and was living in Michigan (MI) since then. He was a construction worker while he was living in Mexico. The patient denied any history of sick contact, animal contact, tobacco use, use of immunosuppressive medications, or a personal or family history of lung cancer.

On examination, vital signs revealed fever with a maximal temperature of 103°F, heart rate of 126 beats/minute, respiratory rate of 24 breaths/min, and blood pressure of 120/70 mmHg. Physical examination was notable for decreased breath sounds over the left middle and lower lung fields associated with dullness to percussion.

Laboratory workup was notable for elevated erythrocyte sedimentation rate (ESR) 77 mm/hour (normal: 0-15 mm/hour) and C-reactive protein (CRP) 20.9 mg/L (normal: 0-5 mg/L). Complete blood count (CBC), liver, and renal function tests were unremarkable other than mild thrombocytosis and lymphopenia (Table 1).

**Table 1:** Initial laboratory results of the patient at admission.

Labs	Value	Reference
White blood cell count	10.7	4.0–12.0 × 10^3^/µL
Neutrophils	75.5	49.0–81.0%
Lymphocytes	12.2	14.0–41.0%
Eosinophils	3.5	0.0–6.0%
Red blood cell count	4.67	3.5–5.55 × 10^6^/µL
Hemoglobin	41.6	42.0–49.5%
Platelets	504	150–400 × 10^3^/µL
Liver function test		
Aspartate transaminase	18	10–40 U/L
Alanine transaminase	37	3–45 U/L
Bilirubin	0.8	0.2–1.2 mg/dL
Alkaline phosphatase	205	45–115 U/L
Albumin	3.8	3.6–5.0 g/dL
Protein	7.4	6.0–8.0 g/dL
Renal function test		
Blood urea nitrogen	18	6–23 mg/dL
Creatinine	0.95	0.60–1.40 mg/dL

**Figure 1:**
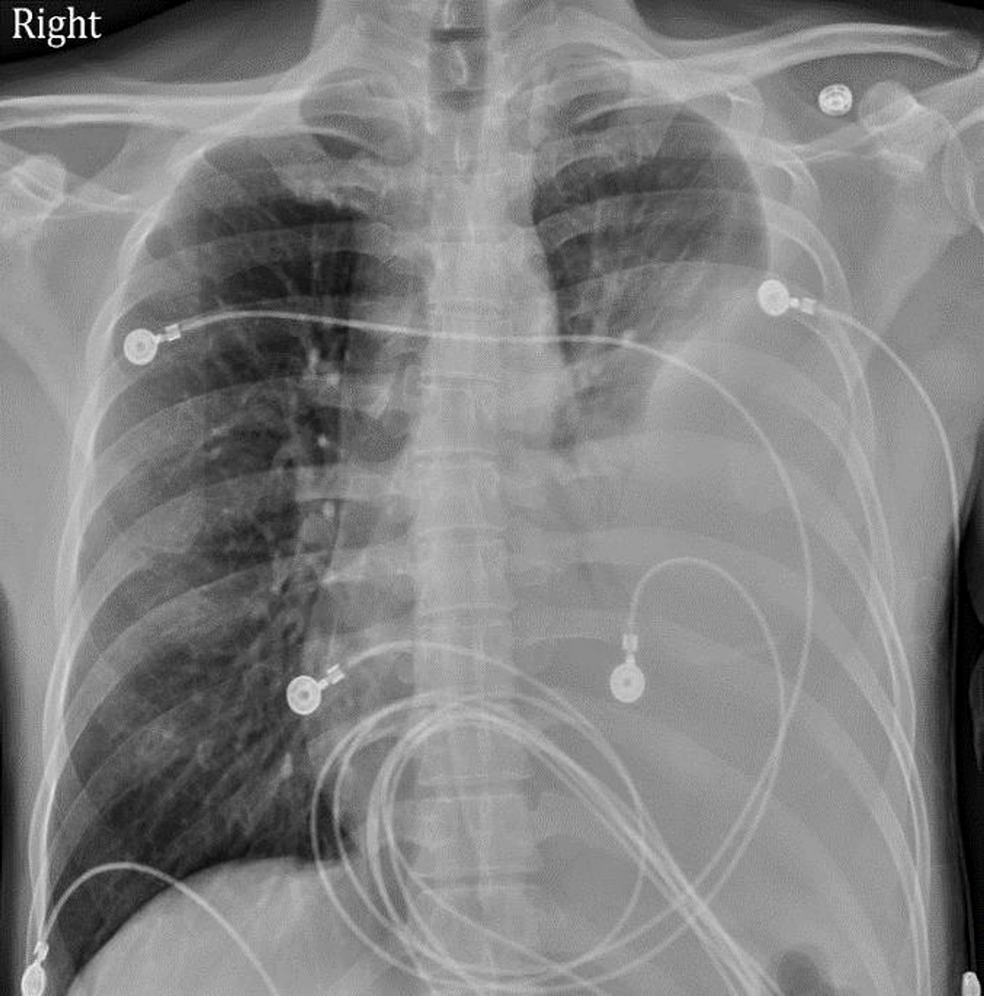
Chest X-ray showing large left pleural effusion with associated airspace disease.

**Figure 2:**
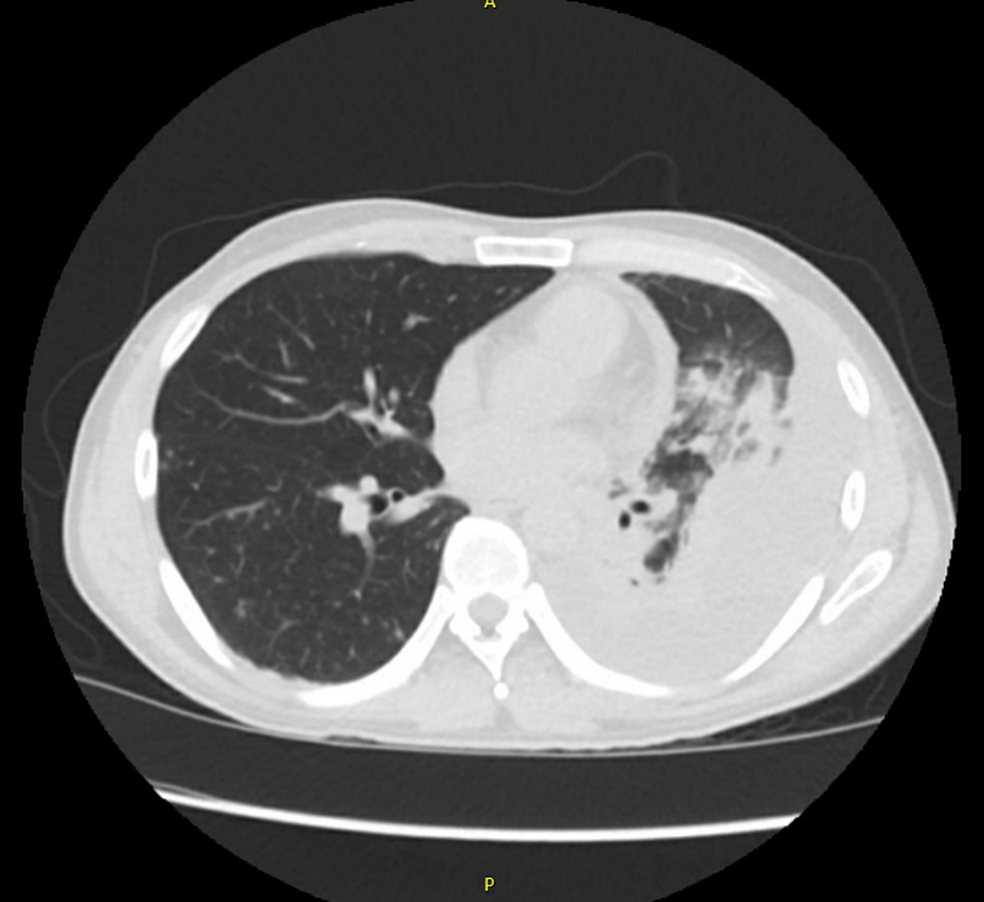
CT chest showing left pleural effusion with associated consolidation.

Chest radiograph showed a large left-sided pleural effusion with adjacent airspace disease (Figure 1). Computed tomography (CT) of the chest with contrast revealed moderate to large left pleural effusion associated with consolidation (Figure 2). CT chest did not reveal any lung nodule, mass, cavity, or lymphadenopathy.

The patient was admitted with an initial diagnosis of complicated lobar pneumonia with associated parapneumonic effusion or empyema. He was started on intravenous antibiotics for community-acquired pneumonia at admission. Thoracentesis was performed and 1,800 mL of serosanguineous pleural fluid was drained. Pleural fluid chemistry was consistent with an exudative process per Light’s criteria (Tables 2, 3). Gram stain and cultures of pleural fluid (bacterial and fungal) did not grow any organisms. Additionally, a cytological examination of the pleural fluid did not show evidence of any neoplastic process.

**Table 2:** Pleural fluid chemistry and microbiology findings.

Pleural fluid chemistry and microbiology	Value
Lactate dehydrogenase, pleural	82 U/L
Protein	4.7 g/dL
Glucose, pleural	103 mg/dL
pH	8
Color	Yellow
Character	Cloudy
Red blood cell count	3,230
White blood cell count	644
Neutrophils	40%
Mononuclear cells	49%
Eosinophils	11%
Gram stain	No organism observed
Gram stain cell count	40–50 white blood cells/hpf
Pleural fluid cultures	No growth

**Table 3:** Serum Lactate dehydrogenase and protein values for Light’s criteria calculation.

Lab	Value	Reference
Lactate dehydrogenase, serum	131 U/L	100–225 U/L
Protein, serum	5.5 g/dL	6.0–8.0 g/dL

Initial microbiological workup was non-diagnostic. Sputum and blood cultures (bacterial and fungal) were non-revealing. Urine antigen tests for *Streptococcus pneumoniae* and *Legionella pneumophila* were negative. The patient tested negative for methicillin-resistant *Staphylococcus aureus* (MRSA), atypical bacterial pathogens (*Mycoplasma pneumoniae*, *Chlamydia pneumoniae*, and *Legionella* sp.), respiratory viruses (Influenza A, Influenza B, respiratory syncytial virus, severe acute respiratory syndrome coronavirus 2) by PCR.

The patient continued to be febrile with persistent respiratory symptoms and was switched to broad-spectrum intravenous antibiotics. Further workup for mycobacterial and fungal etiologies was pursued. QuantiFERON-TB gold test (QFT-G), acid-fast bacilli smear/culture, and pleural fluid adenosine deaminase (ADA) were negative. Initial serological tests including coccidioidal immunoglobulin (Ig)M (coccidioidal precipitin) and IgG (coccidioidal CFor complement-fixing), *Aspergillus *antigen, *Histoplasma *antigen, and *Toxoplasma *IgM were also negative. The human immunodeficiency virus (HIV) antigen-antibody combination assay test was negative.

Bronchoscopy with bronchoalveolar lavage (BAL) and transbronchial lung biopsy was done, which was again non-revealing for neoplastic, mycobacterial, or fungal etiology. BAL cultures continued to show no growth of fungal, bacterial, or mycobacterial organisms.

**Figure 3:**
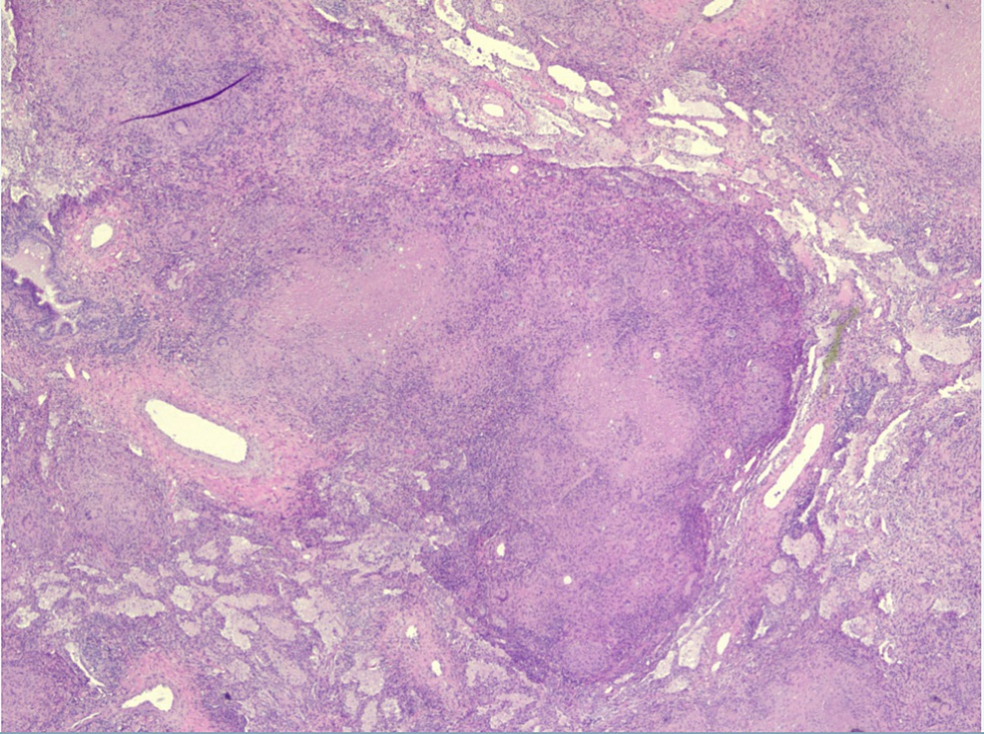
H&E stain of lung tissue showing multiple parenchymal granulomas. H&E: hematoxylin and eosin

**Figure 4:**
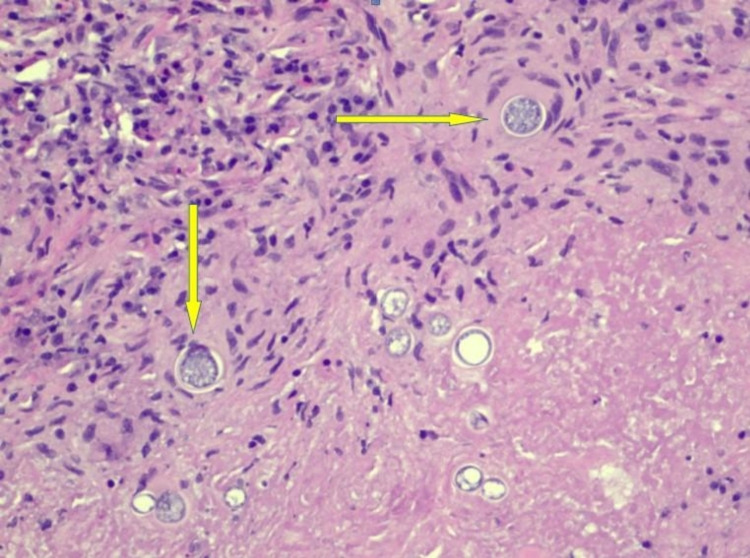
H&E stain showing numerous large round-to-oval spherules of Coccidioides sp. (yellow arrow). H&E: hematoxylin and eosin

**Figure 5:**
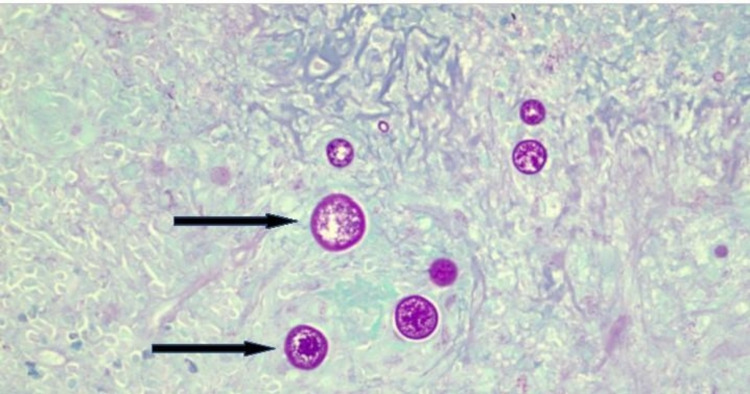
PAS stain showing multiple fungal spherules with endospores (black arrow). PAS: periodic acid-Schiff

**Figure 6:**
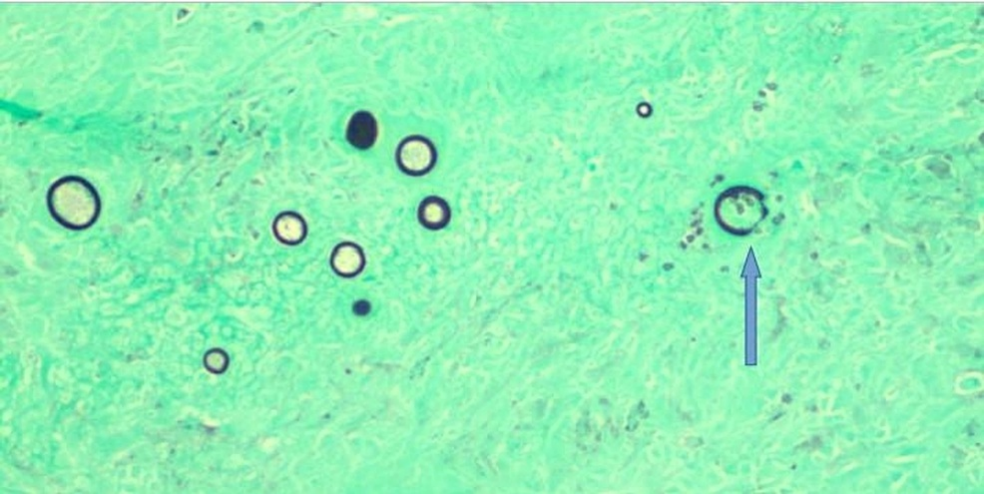
GMS showing fungal spherules with multiple endospores (blue arrow). GMS: Grocott’s methenamine silver stain

Despite being switched to broad-spectrum intravenous antibiotics, the patient did not improve and continued to have fevers and shortness of breath. In the absence of clinical improvement and an unyielding extensive diagnostic workup so far, our multidisciplinary team decided to proceed with open surgical thoracotomy and decortication. Histopathological examination of the lung/pleural specimen from thoracotomy showed extensive necrotizing granulomas associated with reactive fibrosis involving both the lung parenchyma and pleura (Figure 3). In addition to the granulomas, hematoxylin and eosin (H&E) stains revealed numerous large round-to-oval spherules (Figure 4) associated with multinucleate giant cells. Periodic acid-Schiff (PAS) and Grocott’s methenamine silver stain (GMS) further highlighted the spherules filled with endospores, which were diagnostic of coccidioidomycosis (Figures 5, 6). Fungal culture of the lung specimen subsequently grew *Coccidioides immitis*.

Notably, the second set of serum coccidioidal CF antibodies (collected two weeks after the first negative test) returned positive with a reactive titer of 1:64. The second set of coccidioidal precipitin which was also collected after a two-week interval was positive as well.

The patient was started on antifungal treatment with oral fluconazole 800 mg daily. Despite the initiation of fluconazole, the patient continued to be febrile. He also reported worsening diffuse headaches. Subsequent neurological examination and CT brain were unremarkable. A lumbar puncture was done. Cerebrospinal fluid (CSF) analysis (Table 4) revealed the presence of CSF coccidioidomycosis IgG antibody confirming dissemination to meninges.

**Table 4:** CSF findings in the patient. CSF: cerebrospinal fluid; Ig: immunoglobulin

CSF analysis	Results	Reference
Volume	2.0 mL	
Color	Colorless	Colorless
Appearance	Clear	Clear
Red blood cell count	6	0–6/µL
White blood cell count	329	0–5/µL
Neutrophils	8%	0–6%
Mononuclear cells	90%	40–80%
Protein, CSF	105	15–45 mg/dL
Glucose, CSF	41	40–80 mg/dL or >two-thirds of serum glucose
Gram stain	>100 white blood cells/HPF predominately mononuclear. No organisms observed	No organisms
CSF culture	No growth	No growth
Coccidioidal antibody (immunodiffusion)	IgG positive	Negative
Coccidioidal antibody (complement fixation)	IgM negative 1:4	Negative

In the presence of clinical deterioration, fluconazole was switched to intravenous amphotericin B per Infectious Diseases Society of America (IDSA) guidelines. The patient was started on intravenous amphotericin B at a dose of 4 mg/kg q 24 hours. Following the initiation of amphotericin B, the patient improved clinically with subsequent resolution of his fevers. Amphotericin B was discontinued after 10 days, and the patient was re-started on oral fluconazole. Given the meningeal dissemination, the patient was discharged on lifelong azole therapy. The patient has been closely monitored in the outpatient clinic since and has been asymptomatic with a decline of serial antibody titers for the last 20 months. He is also tolerating the azole therapy well without any adverse effects (such as elevated transaminases).

## Discussion

*Coccidioides* sp. (*C. immitis* and *C. posadasii*) is a dimorphic fungus endemic to the southwestern United States, northern Mexico, and certain parts of central and south America. Primary pulmonary coccidioidal infection is caused by the inhalation of coccidioides arthroconidia, which develop into spherical endospores upon entering the host’s lungs. Arthroconidia or fungal spores remain viable in the soil for several years and are released into the air after soil disruption such as during earthquakes or excavation. Therefore, occupations such as farming or construction pose an inherently increased risk. Patients with pulmonary coccidioidomycosis usually have a self-limiting course. Patients with risk factors such as older age (age >65 years), diabetes mellitus, chronic obstructive pulmonary disease, smoking, and immunocompromised state are at risk of developing severe pulmonary complications. Extrapulmonary dissemination is most commonly seen in immunocompromised individuals and accounts for fewer than 1% of all coccidioidal infections.

A pulmonary coccidioidal infection is suspected in a person who is living or has recently traveled to an endemic region in the previous two months. Symptoms usually manifest one to three weeks after exposure or travel. Patients present with influenza or bacterial pneumonia-like symptoms and often have associated constitutional symptoms such as arthralgias, rash (erythema nodosum, erythema multiforme, or generalized exanthem), fatigue, fever, chills, myalgias, headaches, night sweats, and weight loss. Disseminated infection is suspected in a patient with a known immunocompromised state such as HIV/AIDS or taking immunosuppressive agents. The most common sites of dissemination are bones/joints, soft tissues, and meninges. Diagnosis of coccidioidomycosis is confirmed with serology, fungal cultures, PCR, or tissue biopsies [3].

Our patient migrated from the endemic region of Mexico two years ago and had no history of travel since then. He was a construction worker in Mexico and thus at an increased risk of inhaling aerosolized soil. He presented with a left-sided lung consolidation associated with sizeable pleural effusion, which failed to improve with broad-spectrum antibiotics and thoracentesis. We performed an extensive microbiological workup including microscopy and cultures of several samples of sputum, pleural fluid, blood, and BAL which was non-diagnostic. A transbronchial lung biopsy was also performed which did not identify the underlying pathology. The initial serological workup for coccidioidal antibodies by enzyme immunoassay and complement fixation test was also negative. The diagnosis of pulmonary coccidioidomycosis was ultimately confirmed by an open lung biopsy in our patient. We want to emphasize that a surgical lung biopsy is indicated in the workup of unexplained pneumonia when specimen cultures, serology, and thoracoscopic approaches have failed to establish a diagnosis [3].

Serological tests can be used to detect coccidioidal antibodies such as IgM (coccidiodal precipitin) and IgG (coccidioidal CF or complement-fixing). Serological tests include enzyme immunoassay (EIA), immunodiffusion kit (detect IgM or IgG antibodies), or complement fixation (detect IgG antibodies). Titers of ≥1:4 in serum signify current or recent infection, and high titers (≥1:32) signify an increased likelihood of extrapulmonary dissemination. Antibody titers can be low or negative early in the course of disease prior to seroconversion. Coccidioidal IgM is usually detectable approximately after 7-21 days of illness (day 0 being the onset of symptoms), and coccidioidal IgG (coccidiodal CF) is usually detectable between day 21 and 35 with maximum coccidioidal IgG titer detectable between day 21 and 70 [4]. Thus, in early disease, the serology must be repeated in two to four weeks.

In our patient, the first set of serological tests (coccidiodal IgM and IgG antibodies) were negative but the repeat antibody titers two weeks later returned positive with a reactive IgG titer of 1:64. Our case illustrates the importance of serial monitoring of antibody titers during the course of infection in improving the diagnostic yield.

Coccidioidal meningitis is the most severe form of disseminated infection with high mortality. Untreated, it is universally fatal. The most common presentation is a persistent or worsening headache and may be associated with altered mental status, focal neurological deficits, and gait disturbances (especially tandem gait). The lumbar puncture is the diagnostic method of choice. CSF usually demonstrates pleocytosis (lymphocytic) with decreased glucose, elevated protein, and a positive culture or positive serology. The presence of complement-fixing antibodies (IgG) in CSF is diagnostic of coccidioidal meningitis and is important to know because CSF fungal cultures have a low yield [1]. The presence of CSF antigen is also diagnostic but not widely available. A meningeal dissemination was confirmed in our patient by demonstrating complement-fixing antibodies (IgG) in CSF (Table 4). CSF fungal cultures did not show any growth in our patient.

It is also important to note that serologic studies could be falsely negative in immunocompromised patients [5]. In one study, serological evidence of coccidioides infection was negative in one-third of HIV patients [5]. However, we did not identify an immunosuppressed state in our patient. HIV/AIDS, diabetes mellitus, or the use of immunosuppressive agents was not identified in our patient. Additionally, he had a normal neutrophil count and albumin:globulin ratio (Table 1).

IDSA recommends treating coccidioidal meningitis with an azole agent [6]. In the presence of clinical deterioration, therapy can be switched to amphotericin B with or without continuing the azole. Duration of treatment is currently recommended to be lifelong due to the significant morbidity and mortality of relapse. Patients are recommended to be closely monitored for clinical and serological signs of relapse.

## Conclusions

Coccidioidomycosis may have a delayed presentation and should be suspected in patients with respiratory illness even when the history of exposure to the endemic region is remote (more than two months). An open lung biopsy is indicated to establish the diagnosis when specimen cultures, serology, and thoracoscopic approaches have been non-diagnostic. Serial monitoring of serological tests should be done as they may be falsely negative in early disease or immunosuppression. A disseminated illness, though more common with immunosuppression, can also be seen in immunocompetent individuals and should be suspected when signs and symptoms of extrapulmonary involvement are present. Meningeal dissemination is the most severe disseminated form and is associated with high morbidity and mortality. Patients with central nervous system dissemination mostly require lifelong azole therapy along with close clinical and serological monitoring for relapse.
